# Experimentation with and knowledge regarding water-pipe tobacco smoking among medical students at a major university in Brazil*, **

**DOI:** 10.1590/S1806-37132014000200002

**Published:** 2014

**Authors:** Stella Regina Martins, Renato Batista Paceli, Marco Antônio Bussacos, Frederico Leon Arrabal Fernandes, Gustavo Faibischew Prado, Elisa Maria Siqueira Lombardi, Mário Terra-Filho, Ubiratan Paula Santos

**Affiliations:** Pulmonary Division, Heart Institute, University of São Paulo School of Medicine Hospital das Clínicas, São Paulo, Brazil; Department of Pulmonology, University of São Paulo School of Medicine, São Paulo, Brazil; Fundação Jorge Duprat Figueiredo de Segurança e Medicina do Trabalho – FUNDACENTRO, Jorge Duprat Figueiredo Foundation for Occupational Safety and Medicine – São Paulo, Brazil; Pulmonary Division, Heart Institute, University of São Paulo School of Medicine Hospital das Clínicas, São Paulo, Brazil; Pulmonary Division, Heart Institute, University of São Paulo School of Medicine Hospital das Clínicas, São Paulo, Brazil; Pulmonary Division, Heart Institute, University of São Paulo School of Medicine Hospital das Clínicas, São Paulo, Brazil; University of São Paulo School of Medicine, São Paulo, Brazil; Pulmonary Division, Heart Institute, University of São Paulo School of Medicine Hospital das Clínicas, São Paulo, Brazil

**Keywords:** Tobacco products, Smoking, Education, medical, undergraduate, Health knowledge, attitudes, practice

## Abstract

**OBJECTIVE:**

Water-pipe tobacco smoking is becoming increasingly more common among young people. The objective of this study was to estimate the prevalence of the use of water pipes and other forms of tobacco use, including cigarette smoking, among medical students, as well as to examine the attitudes, beliefs, and knowledge of those students regarding this issue.

**METHODS:**

We administered a questionnaire to students enrolled in the University of São Paulo School of Medicine, in São Paulo, Brazil. The respondents were evaluated in their third and sixth years of medical school, between 2008 and 2013. Comparisons were drawn between the two years.

**RESULTS:**

We evaluated 586 completed questionnaires. Overall, the prevalence of current cigarette smokers was low, with a decline among males (9.78% vs. 5.26%) and an increase among females (1.43% vs. 2.65%) in the 3rd and 6th year, respectively. All respondents believed that health professionals should advise patients to quit smoking. However, few of the medical students who smoked received physician advice to quit. Experimentation with other forms of tobacco use was more common among males (p<0.0001). Despite their knowledge of its harmful effects, students experimented with water-pipe tobacco smoking in high proportions (47.32% and 46.75% of the third- and sixth-year students, respectively).

**CONCLUSIONS:**

The prevalence of experimentation with water-pipe tobacco smoking and other forms of tobacco use is high among aspiring physicians. Our findings highlight the need for better preventive education programs at medical schools, not only to protect the health of aspiring physicians but also to help them meet the challenge posed by this new epidemic.

## Introduction

The water pipe used for smoking tobacco was invented in India during the reign of Emperor Akbar (1556-1605) by a physician named Hakim Abul Fath, who suggested that if tobacco smoke passed through a small receptacle of water before being inhaled it would have fewer ill effects on human health. That historical account might be responsible for the current belief that such a water pipe (now known by various names, including narghile, hookah, shisha, and hubble-bubble) is a less harmful way to smoke tobacco. That belief is reinforced by irresponsible marketing practices. For example, the label of a popular brand of water-pipe tobacco available in southwest Asia and North America states "0% tar and 0.5% nicotine".^(^ ^1^ ^)^ In addition to that false sense of safety, reasons for the worldwide spread of the use of water pipes might include increased awareness of the negative health effects of cigarette smoking and the pleasing social interaction that comes with water-pipe tobacco smoking sessions. However, water-pipe tobacco smokers usually share the same mouthpiece (passing it from person to person), which can facilitate the spread communicable diseases, such as colds, respiratory infections, tuberculosis, hepatitis, and herpes. There have been reports of drug-resistant tuberculosis being transmitted via water-pipe tobacco smoking.^(^ ^2^ ^-^ ^5^ ^)^

Because of the quick-light charcoal used in water-pipe tobacco smoking, the average carbon monoxide-nicotine ratio in water-pipe smoke is 50:1, compared with 16:1 in cigarette smoke.^(^ ^6^ ^)^ Among water-pipe tobacco smokers, there have been reports of carbon monoxide poisoning, manifesting as headache, dizziness, nausea, and weakness, followed by syncope.^(^ ^7^ ^)^ The truth is that water-pipe smokers are exposed to many hazardous substances. In 2010, Akl et al.^(^ ^8^ ^)^ conducted a systematic review of 24 studies of the health effects of water-pipe tobacco smoking. The authors found that water-pipe tobacco smoking was significantly associated with lung cancer (OR = 2.12; 95% CI: 1.32-3.42) and respiratory illness (OR = 2.3; 95% CI: 1.1-5.1).

One critical point is that the smoking control community will need to counter the current erroneous argument that water-pipe tobacco smoking has fewer ill effects on human health than does cigarette smoking. In 2009, the state of São Paulo, Brazil, enacted Law no. 13779, which prohibited the sale of water pipes to minors (individuals < 18 years of age).^(^ ^9^ ^)^ Nevertheless, there is a need for additional public health campaigns advising water-pipe tobacco smokers of the health risks to which they are exposing themselves.

As aspiring physicians, medical students might eventually play an important role in shaping smoking control policies. Therefore, it is important that such students are aware of the myths and realities regarding the use of the water pipes. However, there have been few studies of the prevalence of water-pipe tobacco smoking among medical students. In addition, there are few data related to the knowledge, beliefs, and attitudes of such students regarding this subject.

The purpose of this study was to estimate the prevalence of experimentation with water-pipe tobacco smoking, as well as with other forms of tobacco use, including cigarette smoking, cigar/cheroot smoking, pipe smoking, and the use of smokeless tobacco products (chewing tobacco and snuff), among third- and sixth-year medical students. An additional objective was to evaluate attitudes, beliefs, and knowledge of those students regarding the various forms of tobacco use.

## Methods

Medical students at the *Faculdade de Medicina da Universidade de São Paulo* (FMUSP, University of São Paulo School of Medicine) were asked to complete a structured questionnaire regarding their smoking habits. The questionnaire was composed of questions from the Global Health Professions Student Survey^(^ ^10^ ^)^ and additional modules. The respondents were third- and sixth-year students who were present during regular medical school classes. The questionnaire was administered to students in the second semester of their third year and to the same class of students in the second semester of their sixth year. Three classes of students were evaluated: those in their third year in 2008 and in their sixth year in 2011; those in their third year in 2009 and in their sixth year in 2012; and those in their third year in 2010 and in their sixth year in 2013. The questionnaire was completed on a voluntary basis, and all participating students gave written informed consent. The study was approved by the Research Ethics Committee of the FMUSP *Hospital das Clínicas*.

**Chart 1:**
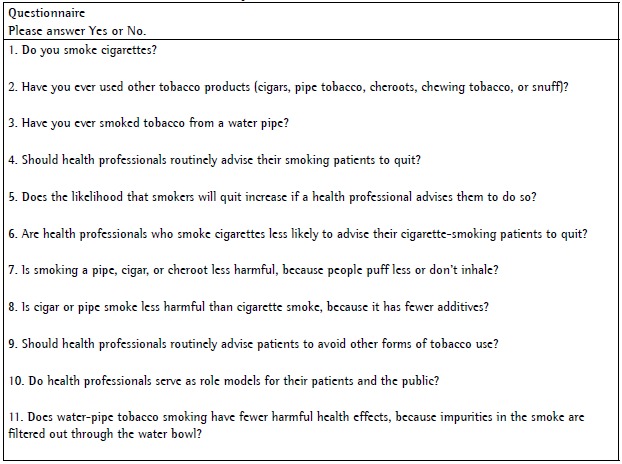
Questionnaire used in the study

Water-pipe tobacco smoking and other forms of tobacco smoking were defined as ever having taken at least a few puffs. Students who had smoked 100 or more cigarettes in their lifetime and were currently smoking were classified as cigarette smokers. Chart 1 shows the questionnaire used in the present study.

Descriptive statistics were calculated. We used Pearson's chi-square test or Fisher's exact test for statistical analyses comparing the proportions of positive responses between the two medical school years. Values of p < 0.05 were considered statistically significant. Data were analyzed with the Statistical Analysis System, version 9.2 (SAS Institute Inc., Cary, NC, USA).

## Results

We evaluated 586 questionnaires, completed by third-year medical students (n = 335) and sixth-year medical students (n = 251). In medical schools in Brazil, the sixth year is the clinical internship year. During that phase, classes are held less often, which could explain the relatively low number of respondents among the sixth-year students.

The mean ages of the third-year and sixth-year students were 22.0 ± 2.76 years and 24.0 ± 1.94 years, respectively. The prevalence of cigarette smoking was significantly higher among male medical students in their third year than among their sixth-year counterparts (Table 1). Table 1 shows that other forms of tobacco use were significantly more common among male students than among female students, in both of the medical school years evaluated (p < 0.0001 for both years). Experimentation with water-pipe tobacco smoking was also more common among the male students (Table 1). The overall prevalence of cigarette smoking among the respondents was quite low and was even lower among the female respondents. The form of tobacco use for which the prevalence was highest was water-pipe smoking (47.32% and 46.75% among the third- and sixth-year students, respectively). As can be seen in Table 1, approximately 40% and 53% of the female and male students, respectively, had experimented with water-pipe tobacco smoking by their third year of medical school (p < 0.005 between the genders).

**Table 1:** Prevalence of the various forms of tobacco use among medical students in their third year (in 2008, 2009, or 2010) and sixth year (in 2011, 2012, or 2013), by gender.a

Form of tobacco use	Third-year medical students		p*	Sixth-year medical students		p*
	Females	Males		Females	Males	
	n = 146	n = 189		n = 114	n = 137	
	n/N (%)	n/N (%)		n/N (%)	n/N (%)	
Cigarette smoking^b^	2/140 (1.4)	18/184 (9.8)	< 0.001	3/113 (2.7)	7/133 (5.3)	ns
Cigar, pipe, or cheroot smoking,^c^ together with tobacco chewing or snuff dipping	16/146 (11.0)	56/189 (30.0)	< 0.0001	13/114 (11.4)	46/137 (33.6)	< 0.0001
Water-pipe tobacco smoking^c^	58/146 (40.0)	101/189 (53.4)	< 0.005	46/113 (40.7)	70/137 (51.0)	ns

n/N : positive responses/total respondents

According to their responses on the questionnaire, all of the students who were cigarette smokers in their third or sixth year of medical school believed that health professionals should advise their patients who smoke to quit smoking. Table 2 shows that the majority of the respondents believe that the likelihood of smokers quitting increases if they are advised to do so by health professionals. However, most of aspiring physicians who were cigarette smokers were not advised to quit by a health professional: 15 (79%) of the 19 smokers evaluated in their third year; and 8 (89%) of the 9 smokers evaluated in their sixth year.

**Table 2:** Attitudes, beliefs, and knowledge regarding cigarette smoking held by medical students in their third year (in 2008, 2009, or 2010) and sixth year (in 2011, 2012, or 2013), by cigarette smoking status.a

Question	Third-year medical students		p*	Sixth-year medical students		p*
	Smokers^b^	Nonsmokers		Smokers^b^	Nonsmokers	
	n = 20	n = 324		n = 10	n = 237	
	n/N (%)	n/N (%)		n/N (%)	n/N (%)	
Should health professionals routinely advise their smoking patients to quit?	20/20 (100)	292/304 (96.0)	ns	10/10 (100)	234/237 (99.0)	ns
Does the likelihood that smokers will quit increase if a health professional advises them to do so?	18/20 (90.0)	273/300 (91.0)	ns	10/10 (100)	222/236 (94.0)	ns
Are health professionals who smoke cigarettes less likely to advise their cigarette-smoking patients to quit?	6/20 (30.0)	194/301 (64.4)	< 0.005	5/10 (50.0)	166/232 (71.5)	ns

n/N : positive responses/total respondents

Health care professionals who smoke cigarettes are less likely to advise their cigarette-smoking patients to quit-that was the belief of 64.5% and 71.6% of the non-cigarette smoking medical students in their third and sixth years, respectively. However, among the smoking students, the proportion of who believed that health professionals who smoke cigarettes are less likely to advise their cigarette-smoking patients to quit increased from 30% in the third year to 50% in the sixth year (Table 2).

Table 3 shows that only a minority of the respondents believed that cigar, pipe, and cheroot smoking is less harmful because smokers puff less or do not inhale. Among the third-year students evaluated, the erroneous belief that cigar and pipe smoking is less harmful because the tobacco involved has lower concentrations of additives was held by 8.33% and 19.01% of the ever-users and never-users of tobacco products other than cigarettes and water-pipe tobacco (p < 0.05). The majority of the respondents believed that health professionals should routinely advise their patients not to use any tobacco product (smoked or smokeless).

**Table 3:** Comparison between ever-users or never-users of tobacco products other than cigarettes and water-pipe tobacco, in terms of their attitudes, beliefs, and knowledge regarding such products, among medical students in their third year (in 2008, 2009, or 2010) and sixth year (in 2011, 2012, or 2013).a

Question	Third-year medical students		p*	Sixth-year medical students		p*
	Ever-user^b^	Never-user		Ever-user^b^	Never-user	
	n = 72	n = 263		n = 59	n = 193	
	n/N (%)	n/N (%)		n/N (%)	n/N (%)	
Is smoking a pipe, cigar, or cheroot less harmful, because people puff less or don’t inhale?	6/72 (8.3)	20/263 (7.6)	ns	3/57 (5.2)	6/191 (3.1)	ns
Is cigar or pipe smoke less harmful than cigarette smoke, because it has fewer additives?	6/72 (8.3)	50/263 (19.0)	< 0.05	2/57 (3.5)	20/187 (10.7)	ns
Should health professionals routinely advise patients to avoid other forms of tobacco use?	71/72 (99.0)	248/263 (94.3)	ns	56/59 (95.0)	183/193 (95.0)	ns

n/N : positive responses/total respondents

Table 4 shows that more than 80% of the aspiring physicians evaluated agreed that health professionals occupy a position of leadership and are role models for their patients, as well as for the general population. More than 98% of the respondents knew that impurities in water-pipe tobacco smoke are not filtered out through the water bowl.

**Table 4:** Comparison between ever-smokers or never-smokers of water-pipe tobacco, in terms of their attitudes, beliefs, and knowledge about water-pipe tobacco smoking, among medical students in their third year (in 2008, 2009, or 2010) and sixth year (in 2011, 2012, or 2013).a

Question	Third-year medical students		p*	Sixth-year medical students		p*
	Ever-smoker^b^	Never-smoker		Ever-smoker^b^	Never-smoker	
	n = 159	n = 176		n = 116	n = 135	
	n/N (%)	n/N (%)		n/N (%)	n/N (%)	
Do health professionals serve as role models for their patients and the public?	131/158 (83.0)	148/176 (84.0)	ns	105/116 (90.5)	119/135 (88.1)	ns
Does water-pipe tobacco smoking have fewer harmful health effects, because impurities in the smoke are filtered out through the water bowl?	2/159 (1.2)	1/175 (0.5)	ns	0/113 (0)	3/131 (2.2)	ns

n/N : positive responses/total respondents

## Discussion

In the present study, the proportions of self-described cigarette smokers among the respondents were lower than those reported for medical students at other universities in Brazil and abroad, as well as being lower than the current estimated prevalence in the general population of Brazil.^(^ ^11^ ^-^ ^14^ ^)^ The National Survey of Health and Nutrition conducted in Brazil in 1989 among smokers > 15 years of age and the Telephone-based System for the Surveillance of Risk and Protective Factors for Chronic Diseases in the population >18 years of age in 27 Brazilian cities showed that public policies for smoking control led to a drop in the prevalence of smokers from 34.8% in 1989 to 12.0% in 2012, corresponding to a 65.51% decrease.^(^ ^13^ ^,^ ^15^ ^)^ It is noteworthy that, in the present study, most of the medical students who smoked reported that they were never advised to quit by a health professional. Medical schools have an ethical responsibility not only to educate but also to raise awareness of health hazards and provide treatment to protect the health of their students.

We found it surprising that, among the medical students evaluated here, experimentation with other forms of tobacco use, such as cigar, pipe, and cheroot smoking, was more common than was cigarette smoking. Experimentation with water-pipe tobacco smoking was critically high among the aspiring physicians at FMUSP, males and females alike. A review of studies on the prevalence of experimentation with water-pipe tobacco smoking among medical students showed that our result (47.0%) is similar to those reported for medical schools in England (51.7%),^(^ ^16^ ^)^ Canada (40%),^(^ ^17^ ^)^ and South Africa (43.5%),^(^ ^18^ ^)^ whereas it is higher than that reported for a medical school in Turkey (28.6%).^(^ ^19^ ^)^

Water-pipe tobacco smoking is the first new tobacco trend of the 21st century.^(^ ^20^ ^)^ It is spreading around the world, having become as fashionable as cigars were in the last century, especially among young professionals and college students. ^(^ ^20^ ^)^ According to Morton et al. (2013), the self-reported prevalence of water-pipe tobacco smoking is highest among the male population of Vietnam (13.02%) and the female population of Russia (3.19%), whereas it remains low in Brazil (0.18% and 0.1% among men and women, respectively). ^(^ ^21^ ^)^ Another possible reason for the spread of water-pipe tobacco smoking is the success of programs to prevent initiation of the (cigarette) smoking habit and to encourage (cigarette) smoking cessation, in Brazil and worldwide. As a result of such anti-smoking campaigns, which target cigarette smokers, susceptible individuals have opted for or migrated to other forms of tobacco use, especially water-pipe smoking.^(^ ^22^ ^)^

The way in which a water pipe is smoked is completely different from the way in which a cigarette is smoked. With a water pipe, smokers inhale combustion products from the charcoal used in heating the tobacco as well as the tobacco smoke itself; the smoke is cooled and appears smoother and easier to inhale because it passes through the water bowl. The way in which an individual smokes a water-pipe (frequency of puffing, depth of inhalation, and length of the smoking session) affects the concentrations of toxins absorbed by the smoker. For example, in a typical (one hour) water-pipe tobacco smoking session, a smoker can inhale 100-200 times the volume of smoke inhaled from a single cigarette.^(^ ^1^ ^)^

Water pipes use a special type of tobacco that is moistened, and there are various flavors and aromas available, such as apple, mint, cherry, chocolate, coconut, licorice, cappuccino, and watermelon.^(^ ^1^ ^,^ ^2^ ^)^ These chemical additives are used by tobacco manufacturers to alter the flavor, and some of them reduce the degree of throat irritation, making the tobacco smoke smoother. That has great appeal to that encourages experimentation by young people, the target population of tobacco industry marketing.^(^ ^6^ ^,^ ^20^ ^)^

The Brazilian *Agência Nacional de Vigilância Sanitária* (ANVISA, National Health Surveillance Agency) regulatory standard designated RDC No. 14 (established 15 March, 2012) bans the use of additives in all tobacco products marketed in Brazil after March 2014. This resolution is an important public health policy measure. By reducing the attractiveness of tobacco, the risk of smoking initiation by children and youths is expected to decrease.^(^ ^23^ ^)^ However, the tobacco industry responded by claiming that 121 additives are essential to the manufacturing process. Consequently, ANVISA created an exception and exempted those additives for a period of 12 months.^(^ ^24^ ^)^ During the same period, ANVISA enacted Ordinance No. 1980, which established a working group of experts on food additives, toxicology, pharmacy, cancer, and tobacco control, who were charged with analyzing all of the additives on the list.^(^ ^25^ ^)^

Our finding that many smoking and nonsmoking medical students believe that health professionals who smoke cigarettes are less likely to advise their cigarette-smoking patients to quit is supported by the results of other studies. ^(^ ^26^ ^,^ ^27^ ^)^ In our sample, a minority of the ever-users of tobacco products other than cigarettes and water-pipe tobacco among the third-year students wrongly believed that tobacco products such as cigars, pipe tobacco, and cheroots have fewer additives and therefore are less harmful than are cigarettes. Only a few students mistakenly believed that the impurities of water-pipe tobacco smoke are filtered out through the water bowl. In the study conducted in Canada,^(^ ^17^ ^)^ medical students (2.5% and 0.6% of the smokers and nonsmokers, respectively) were also found to hold erroneous beliefs, such as the belief that water-pipe tobacco smoke is less harmful than is cigarette smoke. In the study conducted in Turkey,^(^ ^19^ ^)^ which evaluated medical and non-medical university students, the authors found that 65.2% of the smokers and 31.0% of the nonsmokers wrongly believed that water-pipe tobacco smoke is less addictive than is cigarette smoke. These populations are at risk because of a lack of knowledge. Therefore, this issue needs to be more widely discussed at universities and should be publicized through the dissemination of public policies on tobacco control.

We found it surprising that, although nearly all of the respondents in our study knew that water-pipe tobacco smoking is harmful, nearly half had experimented with it. The misconceptions that there is no harm in smoking a water pipe occasionally, that it is a safe form of tobacco use, and that the risk of dependence is low, are common among water-pipe tobacco smokers. However, there is now considerable evidence to the contrary.^(^ ^28^ ^)^ A study conducted in Egypt showed that water-pipe tobacco smokers meet the same criteria for nicotine dependence as do cigarette smokers.^(^ ^29^ ^)^ Another study employed a 10-item version of the Lebanon Waterpipe Dependence Scale to evaluate adult males who were water-pipe smokers in the United Kingdom.^(^ ^30^ ^)^ The authors demonstrated that, among such smokers, the risk factors for water-pipe tobacco dependence included being of Arab ethnicity; having a low level of education; having been alone in the last session of smoking; the last session of smoking having been in the home, in a café, or with friends; smoking sessions being longer in duration; and smoking on a daily basis. The diagnostic criteria for nicotine dependence were met by 47% of sample studied by those authors.^(^ ^30^ ^)^

In the present study, the form of tobacco use for which the prevalence was highest among the FMUSP medical students, regardless of class year and gender, was water-pipe tobacco smoking. However, the prevalence of cigarette smoking was below the national average. The second highest prevalence was found for the use of other tobacco products (smoked and smokeless, excluding cigarettes and water-pipe tobacco), the prevalence of which was higher among the male students than among the female students.

Almost all of our respondents believed that health professionals should advise their smoking patients to quit, and that the likelihood of smokers quitting increases if a health professional provides such advice. More than half of the nonsmoking respondents believed health professionals who smoke cigarettes are less likely to advise their cigarette-smoking patients to quit. Most of the medical students we evaluated were aware of the dangers of using smoked or smokeless tobacco products. They knew that pipes, cigars, and cheroots are no less harmful than are cigarettes because, contrary to popular belief, such products do not in fact have fewer additives and their users do not inhale less smoke. The medical students evaluated also believed that health professionals should routinely advise their patients to quit. In addition, the majority of the respondents believed that health professionals serve as role models, not only for their patients but also for the general population. A minority of the respondents believed that the impurities of water-pipe tobacco smoke are filtered out through the water bowl, which indicates that the majority had an accurate understanding of the harmfulness of water-pipe tobacco smoking. Despite that knowledge, water-pipe tobacco smoking was relatively popular among the FMUSP medical students evaluated.

The data gathered during this study indicate that medical school curricula should focus greater attention on the hazards of (even sporadic) water-pipe tobacco smoking, as well as taking a more effective approach to the myths and realities regarding this form of tobacco use, in order to prevent occasional smokers from becoming regular users. Such measures could be expected to effect a behavioral change among aspiring physicians, reflected in a decrease in the prevalence of water-pipe tobacco smoking. It is also expected that greater knowledge of the issue will make aspiring physicians more confident and motivated to provide routine guidance to their patients, with the objective of preventing all forms of tobacco use and promoting their cessation. Physicians armed with the requisite knowledge will play an important role in controlling the epidemic of water-pipe tobacco smoking.
