# Supplementary material for: Experimentation with and knowledge regarding water-pipe tobacco smoking among medical students at a major university in Brazil
Source: J Bras Pneumol. 2014 Mar-Apr;40(2):102–10. doi: 10.1590/S1806-37132014000200002 (PMC4083634; doi:10.1590/S1806-37132014000200002)
Supplement: Translation

# Translation: Experimentação de e conhecimento sobre narguilé entre estudantes de medicina de uma importante universidade do Brasil*

**Authors:** Stella Regina Martins, Renato Batista Paceli, Marco Antônio Bussacos, Frederico Leon Arrabal Fernandes, Gustavo Faibischew Prado, Elisa Maria Siqueira Lombardi, Mário Terra-Filho, Ubiratan Paula Santos

**Affiliations:** Divisão de Pneumologia, Instituto do Coração, Hospital das Clínicas, Faculdade de Medicina, Universidade de São Paulo, São Paulo (SP) Brasil; Departamento de Pneumologia, Faculdade de Medicina, Universidade de São Paulo, São Paulo (SP) Brasil; Fundação Jorge Duprat Figueiredo de Segurança e Medicina do Trabalho – FUNDACENTRO – São Paulo (SP) Brasil; Divisão de Pneumologia, Instituto do Coração, Hospital das Clínicas, Faculdade de Medicina, Universidade de São Paulo, São Paulo (SP) Brasil; Divisão de Pneumologia, Instituto do Coração, Hospital das Clínicas, Faculdade de Medicina, Universidade de São Paulo, São Paulo (SP) Brasil; Divisão de Pneumologia, Instituto do Coração, Hospital das Clínicas, Faculdade de Medicina, Universidade de São Paulo, São Paulo (SP) Brasil; Faculdade de Medicina, Universidade de São Paulo, São Paulo (SP) Brasil; Divisão de Pneumologia, Instituto do Coração, Hospital das Clínicas, Faculdade de Medicina, Universidade de São Paulo, São Paulo (SP) Brasil

**Keywords:** Produtos do tabaco, Hábito de fumar/prevenção & controle, Educação de graduação em medicina, Conhecimentos, atitudes e prática em saúde

## Abstract

**OBJETIVO:**

O fumo de narguilé com tabaco está aumentando entre os jovens. O objetivo deste trabalho foi estimar a prevalência do uso de narguilé e outras formas de consumo de tabaco, incluindo o fumo de cigarros, entre estudantes de medicina, assim como as atitudes, crenças e conhecimento desses alunos sobre esse assunto.

**MÉTODOS:**

Um questionário foi aplicado aos estudantes da Faculdade de Medicina da Universidade de São Paulo. Os entrevistados eram alunos de terceiro e sexto anos entre 2008 e 2013. As respostas foram comparadas entre os dois anos de graduação.

**RESULTADOS:**

586 estudantes responderam ao questionário. A prevalência de fumantes foi baixa, com um declínio entre os homens (9,78% contra 5,26%) e um aumento no sexo feminino (1,43% contra 2,65%) no 3º e 6º ano, respectivamente. Todos os entrevistados acreditavam que profissionais de saúde devem aconselhar os pacientes a parar de fumar. No entanto, a maioria dos estudantes de medicina fumantes não recebeu aconselhamento médico para deixar de fumar. A experimentação de outros produtos derivados do tabaco foi maior entre os homens (p < 0.0001). Apesar do conhecimento de seus efeitos nocivos à saúde, a experimentação de narguilé foi alta (47,32% e 46,75% entre alunos do terceiro e sexto anos, respectivamente.

**CONCLUSÕES:**

A prevalência da experimentação de narguilé com tabaco e de outras formas de uso de tabaco é alta entre os futuros médicos. Nossos achados enfatizam a necessidade de melhores programas de educação preventiva em universidades médicas para proteger a saúde dos futuros médicos e para ajudá-los a enfrentar esse novo desafio epidêmico.

## Introdução

O cachimbo d'água utilizado para o fumo de tabaco foi inventado na Índia durante o reinado do imperador Akbar (1556-1605) por um médico chamado Hakim Abul Fath, que sugeriu que se o fumo do tabaco passasse por um pequeno recipiente com água antes de ser inalado, teria menos efeitos nocivos à saúde humana. Esse relato histórico pode ser responsável pela crença atual de que tal cachimbo d'água (agora conhecido por vários nomes, como narguilé, *hookah*, *shisha* e *hubble-bubble*) é uma forma menos prejudicial de se fumar tabaco. Essa crença é reforçada por práticas de marketing irresponsáveis. Por exemplo, o rótulo de uma marca popular de narguilé disponível no sudoeste da Ásia e na América do Norte afirma que o produto teria "0% de alcatrão e 0,5% de nicotina".^(^ ^1^ ^)^ Além da falsa sensação de segurança, as razões para a propagação mundial do uso de narguilés podem incluir um aumento da consciência dos efeitos negativos do tabagismo (cigarros) sobre a saúde e a interação social agradável que vem com sessões de uso de narguilé. No entanto, fumantes de narguilé geralmente compartilham o mesmo bocal (passando-o de pessoa para pessoa), o que pode facilitar a propagação de doenças transmissíveis, como resfriados, infecções respiratórias, tuberculose, hepatite e herpes. Há relatos de tuberculose resistente a medicamentos transmitida via narguilés.^(^ ^2^ ^-^ ^5^ ^)^

Devido ao uso de carvão de rápido acendimento no uso de narguilé, a razão média entre monóxido de carbono e nicotina na fumaça de narguilé é de 50:1, em comparação com 16:1 na fumaça do cigarro.^(^ ^6^ ^)^ Entre fumantes de narguilé, há relatos de intoxicação por monóxido de carbono, que se manifesta como dor de cabeça, tonturas, náuseas e fraqueza, seguida de síncope.^(^ ^7^ ^)^ A verdade é que os fumantes de narguilé estão expostos a muitas substâncias perigosas. Em 2010, Akl et al.^(^ ^8^ ^)^ realizaram uma revisão sistemática de 24 estudos sobre os efeitos do uso de narguilé na saúde. Os autores descobriram que o uso de narguilé foi significativamente associado com câncer de pulmão (OR = 2,12; IC95%: 1,32-3,42) e doenças respiratórias (OR = 2,3; IC95%: 1,1-5,1).

Um ponto crítico é que a comunidade de controle do tabagismo precisará contrariar o argumento errôneo atual de que o uso de narguilé tem menos efeitos nocivos sobre a saúde humana do que o tabagismo. Em 2009, o estado de São Paulo promulgou a Lei nº 13.779, que proibiu a venda de narguilés para menores de idade (indivíduos < 18 anos).^(^ ^9^ ^)^ No entanto, há uma necessidade de novas campanhas de saúde pública advertindo os fumantes de narguilé sobre os riscos à saúde a que estão se expondo.

Como futuros médicos, os estudantes de medicina podem eventualmente ter um papel importante na definição das políticas de controle do tabagismo. Portanto, é importante que esses alunos estejam conscientes dos mitos e das realidades sobre o uso de narguilés. Entretanto, existem poucos estudos sobre a prevalência de uso de narguilé entre estudantes de medicina. Além disso, são poucos os dados relacionados com o conhecimento, as crenças e as atitudes desses alunos em relação ao assunto.

O objetivo deste estudo foi estimar a prevalência de experimentação do uso de narguilé, bem como de outras formas de uso do tabaco, incluindo cigarros, charutos/cigarrilhas, cachimbos e produtos de tabaco sem fumaça (tabaco para mascar e rapés) entre estudantes de medicina do terceiro e sexto anos. Outro objetivo foi avaliar as atitudes, as crenças e os conhecimentos dos alunos sobre as diversas formas de uso do tabaco.

## Métodos

Estudantes de medicina da Faculdade de Medicina da Universidade de São Paulo (FMUSP) foram convidados a preencher um questionário estruturado sobre seus hábitos de fumar. O questionário foi composto por perguntas provenientes de *Global Health Professions Student Survey**^(^* ^10^ ^)^ e de módulos adicionais. Os entrevistados eram estudantes do terceiro e sexto anos que estavam presentes durante as aulas regulares na faculdade de medicina. O questionário foi aplicado aos alunos no segundo semestre do terceiro ano e na mesma turma de alunos no segundo semestre do sexto ano. Três turmas de alunos foram avaliadas: aqueles em seu terceiro ano em 2008 e em seu sexto ano em 2011; aqueles em seu terceiro ano em 2009 e em seu sexto ano em 2012, e aqueles em seu terceiro ano em 2010 e em seu sexto ano em 2013. O questionário foi respondido de forma voluntária, e todos os alunos participantes assinaram o termo de consentimento livre e esclarecido. O estudo foi aprovado pelo Comitê de Ética em Pesquisa do Hospital das Clínicas da FMUSP.

O uso de narguilé e de outras formas de consumo de tabaco foi definido como ter dado ao menos algumas tragadas em algum momento na vida. Os estudantes que haviam fumado 100 ou mais cigarros em sua vida e que ainda fumavam foram classificados como fumantes de cigarro. O Quadro 1 mostra o questionário utilizado no presente estudo.

**Quadro 1:** Questionário utilizado no estudo.

Foram calculadas estatísticas descritivas. Foi utilizado o teste do qui-quadrado de Pearson ou o teste exato de Fisher para as análises estatísticas comparando as proporções de respostas positivas entre os dois anos de faculdade. Valores de p < 0,05 foram considerados estatisticamente significativos. Os dados foram analisados com o *Statistical Analysis System*, versão 9.2 (SAS Institute Inc., Cary, NC, EUA).

## Resultados

Foram avaliados 586 questionários, preenchidos pelos estudantes de medicina do terceiro ano (n = 335) e estudantes de medicina do sexto ano (n = 251). Nas escolas médicas no Brasil, o sexto ano é o ano de estágio clínico. Durante essa fase, as aulas são realizadas com menor frequência, o que poderia explicar o número relativamente baixo de respondentes entre os alunos do sexto ano.

As médias de idade dos alunos do terceiro ano e do sexto ano foram 22,0 ± 2,76 anos e 24,0 ± 1,94 anos, respectivamente. A prevalência de tabagismo (cigarros) foi significativamente maior entre os estudantes de medicina do sexo masculino em seu terceiro ano do que entre seus colegas do sexto ano (Tabela 1). A Tabela 1 mostra que outras formas de uso do tabaco foram significativamente mais comuns entre os estudantes do sexo masculino do que entre as do sexo feminino em ambos os anos de faculdade avaliados (p < 0,0001 para ambos os anos). A experimentação de narguilé também foi mais comum entre os estudantes do sexo masculino (Tabela 1). A prevalência geral de tabagismo entre os entrevistados foi bastante baixa e foi ainda menor entre os entrevistados do sexo feminino. A forma de uso de tabaco para o qual a prevalência foi mais elevada foi o uso de narguilé (47,32% e 46,75% entre os estudantes do terceiro e sexto anos, respectivamente). Como pode ser visto na Tabela 1, aproximadamente 40% e 53% dos estudantes do sexo feminino e masculino, respectivamente, tinham experimentado fumar narguilé até o terceiro ano da faculdade de medicina (p < 0,005 entre os sexos).

**Tabela 1:** Prevalência das diferentes formas de uso de tabaco entre estudantes de medicina no terceiro ano (em 2008, 2009 ou 2010) e no sexto ano (em 2011, 2012 ou 2013) de curso por gênero.a

Formas de uso de tabaco	Estudantes do terceiro ano		p*	Estudantes do sexto ano		p*
	Feminino	Masculino		Feminino	Masculino	
	n = 146	n = 189		n = 114	n = 137	
	n/N (%)	n/N (%)		n/N (%)	n/N (%)	
Cigarros^b^	2/140 (1,4)	18/184 (9,8)	< 0,001	3/113 (2,7)	7/133 (5,3)	ns
Charutos, cachimbos ou cigarrilhas,^c^ em conjunto com fumo de mascar ou rapés	16/146 (11,0)	56/189 (30,0)	< 0,0001	13/114 (11,4)	46/137 (33,6)	< 0,0001
Narguilé^c^	58/146 (40,0)	101/189 (53,4)	< 0,005	46/113 (40,7)	70/137 (51,0)	ns

Formas de uso de tabaco	Estudantes do terceiro ano		p*	Estudantes do sexto ano		p*
	Feminino	Masculino		Feminino	Masculino	
	n = 146	n = 189		n = 114	n = 137	
	n/N (%)	n/N (%)		n/N (%)	n/N (%)	
Cigarros^b^	2/140 (1,4)	18/184 (9,8)	< 0,001	3/113 (2,7)	7/133 (5,3)	ns
Charutos, cachimbos ou cigarrilhas,^c^ em conjunto com fumo de mascar ou rapés	16/146 (11,0)	56/189 (30,0)	< 0,0001	13/114 (11,4)	46/137 (33,6)	< 0,0001
Narguilé^c^	58/146 (40,0)	101/189 (53,4)	< 0,005	46/113 (40,7)	70/137 (51,0)	ns

n/N : respostas positivas/total de respostas

ns : não significativo

a Variação de alguns denominadores por falta de dados

b Definido como fumo de = 100 cigarros na vida e autorrelato de fumante atual

c Definido como ter dado uma ou mais tragadas em algum momento da vida

* Teste do qui-quadrado de Pearson.

De acordo com as respostas no questionário, todos os alunos que eram fumantes de cigarros no terceiro ou sexto ano da faculdade de medicina acreditavam que os profissionais de saúde deveriam aconselhar seus pacientes fumantes a parar de fumar. A Tabela 2 mostra que a maioria dos entrevistados acreditava que a probabilidade de fumantes pararem de fumar aumenta se eles são aconselhados a fazê-lo por profissionais de saúde. No entanto, a maioria dos futuros médicos que eram fumantes de cigarros não foi aconselhada a parar por um profissional de saúde: 15 (79%) dos 19 fumantes avaliados em seu terceiro ano e 8 (89%) dos 9 fumantes avaliados em seu sexto ano.

**Tabela 2:** Atitudes, crenças e conhecimento sobre tabagismo (cigarros) dos estudantes de medicina no terceiro (em 2008, 2009 ou 2010) e sexto anos (em 2011, 2012 ou 2013) de curso em função do status tabágico.a

Perguntas	Estudantes do terceiro ano		p*	Estudantes do sexto ano		p*
	Fumantes^b^	Não fumantes		Fumantes^b^	Não fumantes	
	n = 20	n = 324		n = 10	n = 237	
	n/N (%)	n/N (%)		n/N (%)	n/N (%)	
Os profissionais de saúde deveriam aconselhar rotineiramente seus pacientes fumantes a parar de fumar?	20/20 (100)	292/304 (96,0)	ns	10/10 (100)	234/237 (99,0)	ns
A probabilidade de um fumante parar de fumar aumenta se um profissional de saúde o aconselha a parar?	18/20 (90,0)	273/300 (91,0)	ns	10/10 (100)	222/236 (94,0)	ns
Os profissionais de saúde que fumam são menos propensos a aconselhar seus pacientes a parar de fumar?	6/20 (30,0)	194/301 (64,4)	< 0,005	5/10 (50,0)	166/232 (71,5)	ns

n/N : respostas positivas/total de respostas

ns : não significativo

a Variação de alguns denominadores por falta de dados

b Definido como fumo de = 100 cigarros na vida e autorrelato de fumante atual

* Teste do qui-quadrado de Pearson.

Os profissionais de saúde que fumam são menos propensos a aconselhar seus pacientes fumantes a parar de fumar - essa era a crença de 64,5% e 71,6% dos estudantes de medicina não fumantes no terceiro e sexto anos, respectivamente. No entanto, entre os estudantes fumantes, a proporção daqueles que acreditavam que os profissionais de saúde que fumam são menos propensos a aconselhar seus pacientes fumantes a parar de fumar aumentou de 30% no terceiro ano para 50% no sexto ano (Tabela 2).

A Tabela 3 mostra que apenas uma minoria dos entrevistados acreditava que o fumo de charutos, cachimbos e cigarrilhas é menos prejudicial porque os fumantes dão menos tragadas ou não inalam a fumaça. Entre os alunos do terceiro ano avaliados, a crença errônea de que fumar charutos e cachimbos seria menos prejudicial porque o tabaco envolvido tem uma menor concentração de aditivos foi declarada por 8,33% e 19,01% dos fumantes e dos não fumantes de produtos de tabaco que não cigarros ou narguilés (p < 0,05). A maioria dos entrevistados acreditava que os profissionais de saúde deveriam rotineiramente aconselhar seus pacientes a não utilizar quaisquer produtos de tabaco (com ou sem produção de fumaça).

**Tabela 3:** Comparação entre usuários eventuais e não usuários de produtos de tabaco que não cigarros ou narguilés em relação a suas atitudes, crenças e conhecimento sobre esses produtos em estudantes de medicina no terceiro (em 2008, 2009 ou 2010) e sexto anos (em 2011, 2012 ou 2013) de curso.a

Perguntas	Estudantes do terceiro ano		p*	Estudantes do sexto ano		p*
	Usuários eventuais^b^	Não usuários		Usuários eventuais^b^	Não usuários	
	n = 72	n = 263		n = 59	n = 193	
	n/N (%)	n/N (%)		n/N (%)	n/N (%)	
Fumar cachimbos, charutos ou cigarrilhas é menos prejudicial porque os fumantes dão menos tragadas ou não inalam a fumaça?	6/72 (8,3)	20/263 (7,6)	ns	3/57 (5,2)	6/191 (3,1)	ns
A fumaça de cachimbo ou de charuto é menos prejudicial porque tem menos aditivos?	6/72 (8,3)	50/263 (19,0)	< 0,05	2/57 (3,5)	20/187 (10,7)	ns
Os profissionais de saúde deveriam aconselhar rotineiramente seus pacientes a evitar outras formas de uso de tabaco?	71/72 (99,0)	248/263 (94,3)	ns	56/59 (95,0)	183/193 (95,0)	ns

n/N : respostas positivas/total de respostas

ns : não significativo

a Variação de alguns denominadores por falta de dados

b Definido como ter eventualmente dado uma ou mais tragadas em um cachimbo, charuto ou cigarrilha; haver mascado fumo alguma vez; ou haver usado rapé alguma vez

* Teste do qui-quadrado de Pearson.

A Tabela 4 mostra que mais de 80% dos futuros médicos avaliados concordavam que os profissionais de saúde ocupam uma posição de liderança e que são modelos de conduta para os seus pacientes e para a população em geral. Mais de 98% dos entrevistados sabiam que as impurezas da fumaça do tabaco pelo uso de narguilé não são filtradas na água da base do narguilé.

**Tabela 4:** Comparação entre usuários eventuais e não usuários de narguilés em relação a suas atitudes, crenças e conhecimento sobre o fumo de narguilé em estudantes de medicina no terceiro (em 2008, 2009 ou 2010) e sexto anos (em 2011, 2012 ou 2013) de curso.a

Perguntas	Estudantes do terceiro ano		p*	Estudantes do sexto ano		p*
	Usuários eventuais^b^	Não usuários		Usuários eventuais^b^	Não usuários	
	n = 159	n = 176		n = 116	n = 135	
	n/N (%)	n/N (%)		n/N (%)	n/N (%)	
Os profissionais de saúde são modelos de conduta para seus pacientes e o público?	131/158 (83,0)	148/176 (84,0)	ns	105/116 (90,5)	119/135 (88,1)	ns
O uso de narguilé tem menos efeitos prejudiciais para a saúde porque as impurezas na fumaça são filtradas através da água na base?	2/159 (1,2)	1/175 (0,5)	ns	0/113 (0)	3/131 (2,2)	ns

n/N : respostas positivas/total de respostas

ns : não significativo

a Variação de alguns denominadores por falta de dados

b Definido como ter eventualmente dado uma ou mais tragadas em um narguilé

* Teste do qui-quadrado de Pearson

## Discussão

No presente estudo, as proporções de fumantes de cigarro por autorrelato entre os entrevistados foram menores do que o encontrado em estudantes de medicina de outras universidades no Brasil e no exterior, além de ser menor do que a atual prevalência estimada na população geral no Brasil. ^(^ ^11^ ^-^ ^14^ ^)^ A Pesquisa Nacional de Saúde e Nutrição realizada no Brasil em 1989 entre fumantes > 15 anos de idade e o inquérito telefônico de Vigilância de Fatores de Risco e Proteção para Doenças Crônicas na população com mais de 18 anos de idade em 27 cidades brasileiras mostraram que as políticas públicas de controle do tabagismo levaram a uma queda na prevalência de fumantes de 34,8% em 1989 para 12,0% em 2012, correspondendo a uma diminuição de 65,51%.^(^ ^13^ ^,^ ^15^ ^)^ É digno de nota que, no presente estudo, a maioria dos estudantes de medicina que fumavam relatou nunca haver sido aconselhada a parar de fumar por um profissional de saúde. As escolas médicas têm uma responsabilidade ética não só de educar, mas também de aumentar a conscientização sobre os riscos à saúde e oferecer tratamento para proteger a saúde de seus alunos.

Foi surpreendente que, entre os estudantes de medicina aqui avaliados, a experimentação de outras formas de uso do tabaco, como charutos, cachimbos e cigarrilhas, foi mais comum do que fumar cigarros. A experimentação de narguilé foi muito elevada entre os futuros médicos da FMUSP de ambos os gêneros. Uma revisão de estudos sobre a prevalência de experimentação de narguilé entre estudantes de medicina mostrou que nosso resultado (47,0%) é semelhante ao relatado em escolas médicas na Inglaterra (51,7%),^(^ ^16^ ^)^ no Canadá (40%),^(^ ^17^ ^)^ e na África do Sul (43,5%),^(^ ^18^ ^)^ sendo maior do que o relatado em uma escola de medicina na Turquia (28,6%).^(^ ^19^ ^)^

O uso de narguilé é a primeira nova tendência de consumo de tabaco do século XXI.(20) Esse uso está se espalhando ao redor do mundo e tornando-se tão elegante como o fumo de charutos no século passado, especialmente entre jovens profissionais e estudantes universitários.^(^ ^20^ ^)^ De acordo com Morton et al. (2013), a prevalência de uso de narguilé por autorrelato é maior na população masculina do Vietnã (13,02%) e na população feminina da Rússia (3,19%), enquanto essa permanece baixa no Brasil (0,18% e 0,10% entre homens e mulheres, respectivamente).^(^ ^21^ ^)^ Outra razão possível para a propagação do uso de narguilé é o sucesso de programas para prevenir a iniciação do fumo de cigarro e o incentivo na cessação do tabagismo (por cigarros) no Brasil e no mundo. Como resultado dessas campanhas antitabagismo, que têm como alvo os fumantes de cigarros, indivíduos suscetíveis optaram ou migraram para outras formas de uso de tabaco, especialmente o narguilé.^(^ ^22^ ^)^

A maneira pela qual se fuma com um narguilé é completamente diferente da forma pela qual se fuma um cigarro. Com um narguilé, os fumantes inalam produtos da combustão do carvão utilizado no aquecimento do tabaco, bem como o próprio fumo do tabaco; a fumaça é resfriada e parece ser mais suave e mais fácil de inalar, pois ela passa através da água. A maneira pela qual um indivíduo fuma com um narguilé (frequência de tragadas, profundidade da inalação e duração da sessão de fumo) afeta as concentrações de toxinas absorvidas pelo fumante. Por exemplo, em uma sessão típica de fumo com narguilé (uma hora), um fumante pode inalar 100-200 vezes a mais o volume de fumaça de um único cigarro.^(^ ^1^ ^)^

Os narguilés são utilizados com um tipo especial de tabaco que é umedecido, e existem vários sabores e aromas disponíveis, tais como maçã, hortelã, cereja, chocolate, coco, alcaçuz, cappuccino e melancia.^(^ ^1^ ^,^ ^2^ ^)^ Esses aditivos químicos são utilizados pelos fabricantes de tabaco para alterar o sabor, e alguns deles reduzem o grau de irritação da garganta, tornando a fumaça mais suave. Isso tem um grande apelo que incentiva a experimentação por parte dos jovens, a população-alvo do marketing da indústria do tabaco.^(^ ^6^ ^,^ ^20^ ^)^

A norma regulamentar da Agência Nacional de Vigilância Sanitária (ANVISA) RDC nº 14 de 15 de março de 2012 proíbe o uso de aditivos em todos os produtos do tabaco comercializados no Brasil a partir de março de 2014. Essa resolução é uma importante medida de política de saúde pública. Ao reduzir a atratividade do tabaco, espera-se que o risco de iniciação do tabagismo por crianças e jovens diminua.^(^ ^23^ ^)^ No entanto, a indústria do tabaco respondeu afirmando que 121 aditivos são essenciais para o processo de fabricação. Consequentemente, a ANVISA criou uma exceção e liberou os aditivos por um período de 12 meses.^(^ ^24^ ^)^ Durante o mesmo período, a ANVISA publicou a Portaria nº 1.980, que criou um grupo de trabalho de peritos em aditivos alimentares, toxicologia, farmácia, câncer e controle do tabaco a fim de analisar todos os aditivos listados.^(^ ^25^ ^)^

Nosso achado de que muitos estudantes de medicina fumantes e não fumantes acreditam que os profissionais de saúde que fumam são menos propensos a aconselhar seus pacientes fumantes a parar de fumar corrobora os resultados de outros estudos.^(^ ^26^ ^,^ ^27^ ^)^ Em nossa amostra, uma minoria de estudantes do terceiro ano que alguma vez experimentou produtos de tabaco que não cigarros ou narguilé erroneamente acreditava que produtos de tabaco, como charutos, tabaco de cachimbo e cigarrilhas, têm menos aditivos e, portanto, são menos prejudiciais do que os cigarros. Apenas alguns estudantes equivocadamente acreditavam que as impurezas da fumaça do tabaco são filtradas através da água na base do narguilé. No estudo realizado no Canadá,^(^ ^17^ ^)^ estudantes de medicina (2,5% e 0,6% dos fumantes e não fumantes, respectivamente) também apresentavam crenças errôneas, como a crença de que o fumo do tabaco com narguilé é menos prejudicial do que o de cigarros. No estudo realizado na Turquia,^(^ ^19^ ^)^ que avaliou universitários de medicina e de outros cursos, os autores constataram que 65,2% dos fumantes e 31,0% dos não fumantes erroneamente acreditavam que a fumaça do narguilé causa menos dependência que a fumaça do cigarro. Essas populações estão em risco por falta de conhecimento. Portanto, esse problema precisa ser mais amplamente discutido nas universidades e deve ser divulgado por meio da disseminação das políticas públicas de controle do tabaco.

É surpreendente que, apesar de quase todos os entrevistados em nosso estudo saberem que o uso de narguilé é prejudicial, quase metade desses já o tinham experimentado. Os equívocos de que não há mal nenhum em fumar narguilé de vez em quando, que essa é uma forma segura de consumo de tabaco e que o risco de dependência é baixo são comuns entre os usuários de narguilé. No entanto, agora há evidências consideráveis do contrário.^(^ ^28^ ^)^ Um estudo realizado no Egito mostrou que usuários de narguilé preenchem os mesmos critérios quanto à dependência da nicotina como os fumantes de cigarros.^(^ ^29^ ^)^ Outro estudo utilizou uma versão de 10 itens da *Lebanon Waterpipe Dependence Scale* para avaliar adultos do sexo masculino que eram usuários de narguilé no Reino Unido.^(^ ^30^ ^)^ Os autores demonstraram que, entre esses fumantes, os fatores de risco para a dependência de tabaco para narguilé incluíam ser de etnia árabe, ter baixo nível educacional, ter fumado sozinho da última vez que fumou, ter fumado em casa, em um café ou com amigos da última vez que fumou, ter sessões de fumo com maior duração e fumar diariamente. Os critérios diagnósticos para a dependência de nicotina foram preenchidos por 47% da amostra estudada por esses autores.^(^ ^30^ ^)^

No presente estudo, a forma de uso do tabaco para o qual a prevalência foi maior entre os estudantes de medicina da FMUSP, independentemente do ano da classe e gênero, foi o uso de narguilé. No entanto, a prevalência de tabagismo (cigarros) foi menor que a média nacional. A segunda maior prevalência encontrada foi o uso de outros produtos do tabaco (com e sem fumaça, excluindo cigarros e narguilé), e tal prevalência foi maior entre os estudantes do sexo masculino do que entre as estudantes do sexo feminino.

Quase todos os nossos entrevistados acreditavam que os profissionais de saúde deveriam aconselhar seus pacientes fumantes a parar de fumar e que a probabilidade de fumantes pararem de fumar aumentaria se um profissional de saúde fornecesse tal conselho. Mais da metade dos entrevistados não fumantes acreditava que profissionais de saúde que fumam cigarros são menos propensos a aconselhar seus pacientes fumantes de cigarro a parar de fumar. A maioria dos estudantes de medicina que avaliamos estava ciente dos perigos do uso de produtos de tabaco com ou sem fumaça. Eles sabiam que cachimbos, charutos e cigarrilhas não são menos prejudiciais que os cigarros porque, ao contrário da crença popular, esses produtos não têm, de fato, menos aditivos, e seus usuários não inalam menos fumaça. Os estudantes de medicina avaliados também acreditavam que os profissionais de saúde deveriam rotineiramente aconselhar seus pacientes a parar de fumar. Além disso, a maioria dos entrevistados acreditava que os profissionais de saúde servem como modelos de conduta não só para os seus pacientes mas também para a população em geral. Uma minoria dos entrevistados acreditava que as impurezas da fumaça do tabaco são filtradas através da água na base do narguilé, o que indica que a maioria tinha uma compreensão exata da nocividade do fumo de narguilé. Apesar desse conhecimento, o uso de narguilé foi relativamente comum entre os estudantes de Medicina da FMUSP avaliados.

Os dados compilados durante o presente estudo indicam que os currículos escolares de medicina deveriam dar maior atenção aos perigos do uso (mesmo esporádico) de narguilés, bem como ter uma abordagem mais eficaz quanto aos mitos e realidades sobre essa forma de uso do tabaco a fim de evitar que fumantes ocasionais tornem-se usuários regulares. Tais medidas podem ter o efeito de mudar o comportamento dos futuros médicos, refletido uma diminuição na prevalência do uso de narguilés. Espera-se também que um maior conhecimento do assunto fará com que os futuros médicos sejam mais confiantes e motivados a fornecer orientações de rotina para seus pacientes a fim de prevenir todas as formas de uso do tabaco e promover a sua cessação. Médicos munidos do conhecimento necessário desempenharão um papel importante no controle da epidemia de uso de narguilés.
